# Multirate Processing with Selective Subbands and Machine Learning for Efficient Arrhythmia Classification †

**DOI:** 10.3390/s21041511

**Published:** 2021-02-22

**Authors:** Saeed Mian Qaisar, Alaeddine Mihoub, Moez Krichen, Humaira Nisar

**Affiliations:** 1 College of Engineering, Effat University, Jeddah 21478, Saudi Arabia; 2 Communication & Signal processing Lab, Energy and Technology Centre, Effat University, Jeddah 21478, Saudi Arabia; 3 Department of Management Information System and Production Management, College of Business and Economics, Qassim University, P.O. Box 6640, Buraidah 51452, Saudi Arabia; a.mihoub@qu.edu.sa; 4 Faculty of CSIT, Al-Baha University, Al-Baha 65731, Saudi Arabia; moez.krichen@gmail.com; 5 ReDCAD Laboratory, University of Sfax, Sfax 3029, Tunisia; 6 Department of Electronic Engineering, Faculty of Engineering and Green Technology, Universiti Tunku Abdul Rahman, Kampar 31900, Malaysia; humaira@utar.edu.my; 7 Centre for Healthcare Science and Technology, Universiti Tunku Abdul Rahman, Kajang 43000, Malaysia

**Keywords:** multirate processing, selective subband coefficients, computational complexity, compression, classification, electrocardiogram (ECG), machine learning, mobile healthcare, wavelet decomposition

## Abstract

The usage of wearable gadgets is growing in the cloud-based health monitoring systems. The signal compression, computational and power efficiencies play an imperative part in this scenario. In this context, we propose an efficient method for the diagnosis of cardiovascular diseases based on electrocardiogram (ECG) signals. The method combines multirate processing, wavelet decomposition and frequency content-based subband coefficient selection and machine learning techniques. Multirate processing and features selection is used to reduce the amount of information processed thus reducing the computational complexity of the proposed system relative to the equivalent fixed-rate solutions. Frequency content-dependent subband coefficient selection enhances the compression gain and reduces the transmission activity and computational cost of the post cloud-based classification. We have used MIT-BIH dataset for our experiments. To avoid overfitting and biasness, the performance of considered classifiers is studied by using five-fold cross validation (5CV) and a novel proposed partial blind protocol. The designed method achieves more than 12-fold computational gain while assuring an appropriate signal reconstruction. The compression gain is 13 times compared to fixed-rate counterparts and the highest classification accuracies are 97.06% and 92.08% for the 5CV and partial blind cases, respectively. Results suggest the feasibility of detecting cardiac arrhythmias using the proposed approach.

## 1. Introduction

The electrocardiogram (ECG) signal contains essential cardiac functionality information [1], therefore, the disorder in heart function can be diagnosed by a precise examination of the ECG [2]. Heart disease is life threatening in nature [3] and early diagnosis and treatment may save lives. The automated diagnosis of arrhythmia is based on the morphological patterns and frequency content of ECG [4,5,6].

The ECG signal may be changed by interference and physiological artifacts, which reduces the efficacy of the automatic diagnostic. Several signal processing techniques have been used to address these limitations, such as wavelet transform [7], adaptive-rate filtering [8], eigenvalue decomposition [9], extended Kalman filtering [10] and Fourier decomposition [11]. To identify attributes that can help in automated diagnosis, the denoised ECG signals are analysed. Tunable-Q wavelet transform (TQWT) [12], wavelet-based kernel principle component analysis [13], orthogonal wavelet filters [14], wavelet packet entropy (WPE) [15], discrete wavelet transform (DWT) [16], short time Fourier transform (STFT) [17], bispectrum [18] and Hilbert transform [19] are several widely utilized feature extraction methods.

The timely diagnosis of arrhythmia conditions enables successful treatment of heart failure. Patients with heart attacks, therefore, need constant monitoring. Hence, wearable ECG sensors are useful in this scenario [20,21]. Continuous recording and analysis of the multi-channel ECG signals is necessary to attain an accurate diagnosis. However, this results in a large amount of data that needs to be processed and analysed. Manual monitoring of such a large amount of data is not feasible. Therefore, automated ECG signal processing and analysis approaches have been proposed [6,7,12,14,15,16,17,22,23,24,25]. In the event of a poorly tolerated rhythm disorder, the type of arrhythmia should be diagnosed quickly in order to start treatment as quickly as possible [6,7,12,14]. Depending on the type of arrhythmia, several medications may be prescribed. Some mild arrhythmia types do not require rapid treatment. Others can be treated with medication. When the case is serious then other approaches can be used, such as pacemakers, cardiac defibrillation, and surgery. However, similarities between different arrhythmia types can make the identification task challenging for the medical experts and also for the automated ECG systems. In this context, sophisticated signal conditioning, feature extraction and classification is commonly used in the automated ECG analysis systems. In [6] Qaisar et al. used autoregressive Burg (ARB) for features extraction. Onward, rotation forest (RoF) is used to recognise arrhythmia based on these extracted features. In [7] Qaisar et al. used wavelet decomposition-based subbands statistical features extraction approach with random forest (RF) for arrhythmia classification. In [12] Jha et al. Used TQWT for features extraction and support vector machine (SVM) for the arrhythmia classification. In [14] Sharma et al. used DWT with fuzzy and Renyi entropy plus the fractal dimension approach for features extraction. They used k-nearest neighbour (KNN) for the classification purpose. In [15], Li and Zhou have used wavelet packet entropy (WPE) for features extraction and RF for classification of arrhythmia. In [16] Gutiérrez-Gnecchi et al. have used the DWT based approach for feature extraction and probabilistic neural network (PNN) for classification. In [17] Huang et al. have used an STFT-based features extraction approach with a convolutional neural network (CNN) for classification. In [22] Sahoo et al. used DWT with temporal and morphological approaches for features extraction. They used SVM for the classification purpose. In [23] Yildirim used DWT for features extraction with long short term memory (LSTM) for classification. In [24] Hou et al. achieved features extraction by using LSTM-based auto-encoder (AE) network. The SVM is used for features extraction. In [25] Anwar et al. achieved features extraction by using a hybrid time-frequency analysis approach. The classification is carried out by using a artificial neural network (ANN).

The realization of cloud-connected wireless ECG wearables is constrained due to strict limits on the volume, weight and power consumption. Hence methods with signal compression [20,21,26] irregular sampling [6,27] and adaptive rate processing [6,7,8] have been conducted in this area. The low computational complexity approaches are required for efficient wearable ECG gadgets and cloud-based mobile healthcare solutions.

In the context of cloud-based mobile healthcare, the aim of this work is to participate in the realization of contemporary power and computationally efficient ECG wearables. The contribution of this paper is proposing a novel and efficient, multirate processing based, automated arrhythmia recognition approach. Moreover, a partial blind protocol is suggested to evaluate the classification performance of the suggested method. The system is realized by intelligently using the multirate ECG signal processing, QRS selection, lower-taps FIR filter-based denoising, particular wavelet decomposition scheme-based feature extraction, frequency content-based subband coefficient selection and robust machine learning techniques. The functioning steps are presented below.

(i). Multirate processing is used for computationally efficient system realization.
(ii). QRS selection is used to focus on the relevant signal part while avoiding the unwanted baseline. It also enhances the system computational effectiveness.
(iii). Each selected QRS segment is filtered by using a multirate lower-taps FIR filter.
(iv). An effective wavelet decomposition scheme is proposed for subband extraction.
(v). A frequency content-dependent subband coefficient selection is performed to attain the dimension reduction.
(vi). The performance of KNN, ANN, SVM, RF, decision tree (DT) and bagging (BG) is studied for the automated recognition of arrhythmia by using the forehand selected features.
(vii). To avoid over fitting and any biasness, the classification performance is evaluated by using the 5CV and a novel partial blind testing protocol.

## 2. Materials and Methods

**Figure 1:**
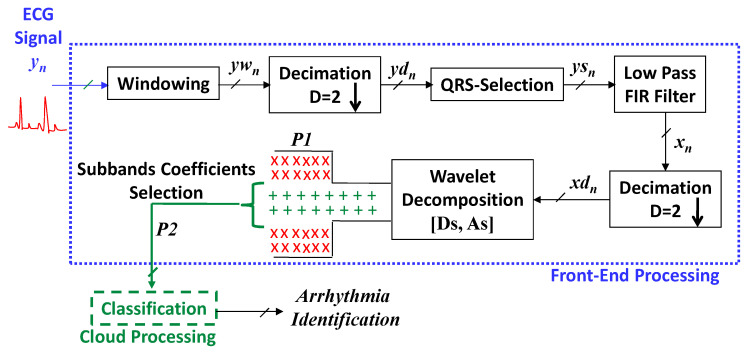
The proposed system block diagram.

Figure 1 demonstrates the proposed structure, where the proposed ECG front-end processing blocks, embedded in wearables, are enclosed in the blue colour ‘....’ border. A green colour ‘---’ border encloses the cloud computing module. The blue colour signal $y_{n}$ is the input from the dataset [28]. The suggested processing steps are presented by the black and green colour signals and blocks.

### 2.1. Dataset

The proposed solution is evaluated on the MIT-BIH Arrhythmia Database (MITDB) available on PhysioNet. The database consists of multi-channel ECG recordings with heartbeats annotated by cardiologists [28,29].

The analogue signal is digitized by using an 11-bit analogue-to-digital converter (ADC), with a sampling frequency of 360 Hz. An antialiasing filter with a cut-off frequency of 60 Hz is used at the input of ADC. In this analysis, based on the availability of data in the desired format, four major forms of arrhythmias are considered. These are normal signals (N), right bundle branch block (RBBB), atrial premature complexes (APC) and left bundle branch block (LBBB). These are clinically important categories of arrhythmia and are frequently used for evaluating the automated arrhythmia classification techniques [6,7,14,15,16,17,22,23,24]. To prevent any biasness, instances of every examined class are collected from multiple 30-min duration ECG records. This allows studying the proposed system performance in case of multiple patients, belongs to both genders male and female, and avoiding any over fitting and biasness. Class N instances are extracted for three different subjects by using the 100, 106 and 112 records and respectively belong to a male of age 69 years, a female of age 24 years and a male of age 54 years. Class RBBB instances are derived from 118, 124 and 212 records and respectively belong to a male of age 69 years, a male of age 77 years and a female of age 32 years. The instances for the APC class are extracted from the 209, 222 and 232 records and respectively belong to a male of age 62 years, a female of age 84 years and a female of age 76 years. The instances for the LBBB class are extracted from the 109, 111 and 214 records and respectively belong to a male of age 64 years, a female of age 47 years and a male of age 53 years. For each class, 510 instances are considered and 170 instances are collected for each intended subject. This resulted in a dataset consisting of 2040 instances.

Classifiable features of each considered ECG instance are extracted by using a specifically designed wavelet-based decomposition scheme (cf. Section 2.3). It generates the subband coefficient feature set *P1*. Onward frequency content-based subband coefficients are selected for an effective dimension reduction and it produces the feature set *P2*. Finally, features set *P1* and *P2* are used as input of the considered classifiers.

### 2.2. Decimation with QRS Selection and Denoising

The functioning of traditional methods for digitizing and analysing ECG signals is time-invariant [7,8]. Due to the static temperament, selection of the worst-case system parameterization is necessary [8]. In the case of time varying ECG signals, this results in an inefficient and computationally-complex realization.

In this framework, the digitized signal $y_{n}$ is firstly divided in fix-length windows, $yw_{n}$. According to the statistics of dataset [28], the average cardiac pulse duration for different arrhythmia is 0.9 s. Therefore, the continuous ECG signals are split in segments of 0.9 s in length, each consisting of $N_{r}\;$ = 324 samples. It is attained by using a rectangular window function [30]. Onward, each $yw_{n}$ is subsampled with a factor of D=2 to obtain $yd_{n}=yw_{Dn}$. Aliasing can be caused by decimation without a prior anti-aliasing filtering [30]. An appropriate selection of D, however, enables caring out the decimation without preliminary filtering (which is computationally complex). In this situation, for this study the value of D should adhere to the condition: $D\leq \frac{F_{S}}{F_{Nyq}}=3$. Here, $F_{S}=360\;H$z, $F_{Nyq}=2.$ $f_{max}$ and $f_{max}=60\;H$z is the bandwidth of $y_{n}\;$ [28]. It demonstrates that for the selected D=2, decimation does not induce aliasing.

**Figure 2:**
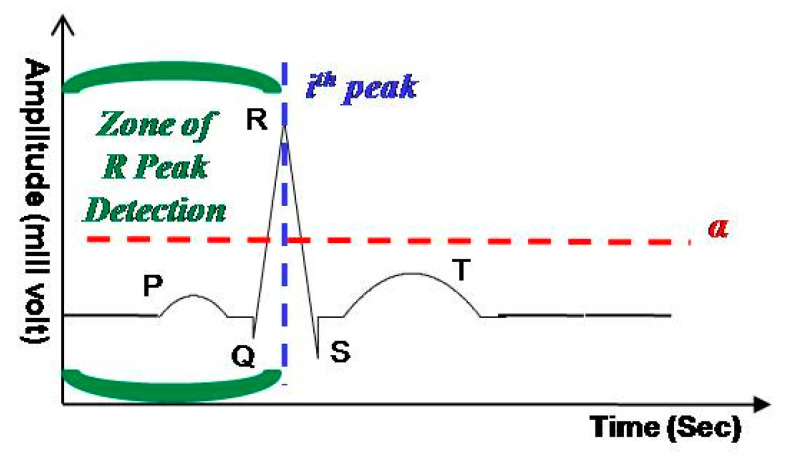
The principle of QRS selection.

A heartbeat has three basic components, namely, QRS complex, T wave and P wave (cf. Figure 2). The QRS complex contains pertinent information about the cardiac system functionality and is used for the identification of arrhythmia [25,31]. Therefore, the QRS selection process is performed on $yd_{n}$ to extract the QRS complexes, $ys_{n}$. The process is based on the principle of detecting R peaks and selecting a segment of 60 samples around the R peak. The choice of 60 samples per segment is made to assure a proper segmentation of QRS. According to [28], for different arrhythmia classes the QRS complex width is limited to 250 ms. Therefore, the selection of 60 samples, around the R peak, at a sampling rate of 180 Hz results in a segment length of 333 ms. This assures a proper QRS complex selection for the considered dataset.

The online selection of QRS complexes was implemented by thresholding the ECG signal with a threshold α. The value of α is selected equal to 50% of the average R peak amplitudes in the considered dataset [28]. The process is depicted with the help of Figure 2. It shows that the incoming samples series of $yd_{n}$ is compared with α and on the detection of α crossing the *i*th R peak is located by picking the maximum sample value on the leading edge of R pulse. If the windowed and decimated signal crosses α then the *i*th QRS is segmented and processed. On other hand, it is ignored and the upcoming α crossing is considered as the *i*th QRS complex. The process continues for each window.

A low-pass finite impulse response (FIR) filter based on the Parks–McClellan algorithm is used for denoising resulting in the removal of the muscle artifacts and power line interference from the ECG signal [8]. The outcome of denoising is the efficient decomposition of subbands and improved feature extraction and classification. The valuable spectrum of the ECG signal is limited to 40 Hz [6]. Hence, a low-pass linear phase FIR filter with a cut-off frequency, $F_{C}\;$ = 40 Hz, and sampling frequency of 180 Hz is implemented offline. For correct filtering, the sampling frequency of the incoming signal and filter must be coherent [30]. In addition, $F_{C}$ should be less than half of the sampling frequency of the signal [30].

The FIR filter orders are proportional to the operating sampling frequency for the design parameters used, such as cut-off frequency, transition-band width, and pass-band and stop-band ripples [32]. Therefore, the previous subsampling enables the denoted signal $x_{n}$ to be efficiently achieved. It is by halving the number of samples to be processed firstly, and secondly by using roughly a half-order FIR filter compared to the counter equals that are built to work at $F_{S}=360\;\mathrm{Hz}$ for the same specifications.

$x_{n}$ is further subsampled with D=2 to obtain $xd_{n}=x_{Dn}$. The choice of D=2, for the studied case, adheres to the condition: $D\leq \frac{Fs1}{F_{Nyq}}=2.25$ and, thus, does not induce aliasing. Here, Fs1=180 Hz, $F_{Nyq}=2.f_{maxf}$ and $f_{maxf}$ is the bandwidth of $x_{n}$ and is equal to $F_{C}=40\;\mathrm{Hz}$. This cascading step of second-level subsampling further decreases the computational complexity of the system by reducing the amount of information to be processed by the decomposition and dimension reduction stages.

### 2.3. Wavelet Decomoposition

The multi-resolution time-frequency examination of the non-stationary signals could result in an efficient extraction of features [12,15,16,22,23]. In this context, the Wavelet Transform (WT) is commonly adopted. It could be represented mathematically by Equation (1), here s and u correspond to the dilation and the translation parameters respectively:

(1) $$W_{x}^{\psi }\left(u,s\right)=\;\frac{1}{\sqrt{S}}\int_{-\infty }^{+\infty }x\left(t\right)\psi *\left(\frac{\left(t-u\right)}{s}\right)dt$$

**Figure 3:**
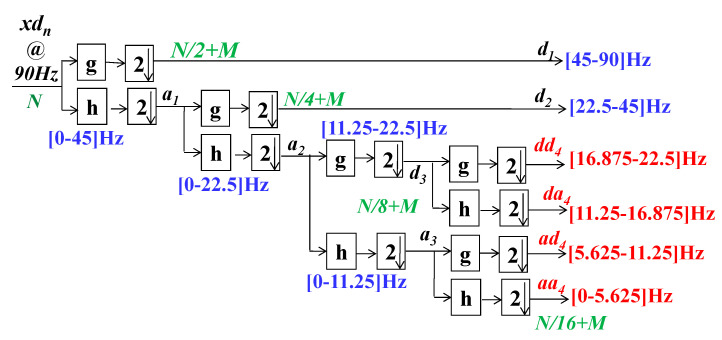
The employed wavelet decomposition scheme.

For digital signal examination the DWT (discrete time wavelet transform) is adopted. Using half-band digital filters with subsampling with a factor of two, the decomposition of subbands is achieved. Thus, in the proposed system each segment $xd_{n}$ is decomposed by using the Daubechies algorithm-based wavelet. This makes it possible to extract the subband coefficients, specifically approximation, $\;a_{m}$ and detail, $d_{m}$, coefficients at each level, m. This process could be formulated mathematically by using Equations (2) and (3). A fourth level of decomposition is achieved. Thus, m∈{1, 2,3,4}. $g_{2n-k}$ and $h_{2n-k}$ are, respectively, the half-band low-pass and high-pass filters of length *M* + 1. Figure 3 further clarifies this process.

(2) $$a_{m}=\sum_{k=1}^{K_{g}}xd_{n}.g_{2n-k}.$$

(3) $$d_{m}=\sum_{k=1}^{K_{g}}xd_{n}.h_{2n-k}.$$

### 2.4. Subband Coefficient Selection

According to studies presented in [25,33], it is clear that for arrhythmia classification the ECG band of interest is between [0; 22] Hz. Therefore, the subband coefficients from dd4, da4, ad4 and aa4 are selected. Each considered subband is composed of eight coefficients. Therefore, in total, 32 features were employed to represent each selected segment. Each selected segment is considered as an instance. To confirm the effectiveness of the subband coefficient selection the classification performance is studied on two features set. The first feature set is composed of all subbands, d1, d2, dd4, da4, ad4 and aa4 coefficients. It results in 62 features per instance and results in a feature set *P*1 = 2040 × 62 for all considered four-class arrhythmia instances. The second feature set is composed of selected subbands, dd4, da4, ad4 and aa4. It results in a feature set *P*2 = 2040 × 32 for all considered four-class arrhythmia instances.

### 2.5. Classification Methods

The classification algorithms used in this study are described in the following subsections. Algorithms were implemented to predict the right class for each datasets sample. Before classification, a min-max scaler was applied to normalize feature ranges between 0 and 1. This operation is recommended and even mandatory for some classification methods. Please note that all pre-processing, training, and testing operations were implemented using Python and well-known data science packages, such as Pandas and Scikit-learn. Empirical results were computed using a 16GB-RAM PC disposing of an Intel^®^ Core™ i7-8550U Processor. Hyperparameters tuning steps were explored to select optimal hyperparameters for each classification algorithm and to prevent overfitting issues.

#### 2.5.1. Artificial Neural Network (ANN)

ANNs are based on the concept of biological neural networks [34]. An artificial neuron is the basic building block for ANN. ANNs rely on three simple operations which are multiplication, summation and activation. Hence, ANNs are a set of linked input and output units. The learning is accomplished by adjusting the weights of each link which results in the classification of test data. The inherent parallel nature of ANNs can be used to speed up the classification process.

The main advantage of ANNs is that they can learn and model the non-linear and complex relationships among inputs and outputs. However, ANNs require large amounts of data as the number of hidden layers increases.

In this case, standard multilayer perceptron (MLP) approach is adopted. Concretely, two hidden layers—containing each almost 50 nodes for the reduced setting, and 90 nodes for the full setting—have shown to be the best topology for the MLP architecture.

#### 2.5.2. k-Nearest Neighbours (k-NN)

k-NN is a simple classification method based on assumption that similar things are located close to each other. Hence, k-NN looks for k number of samples (also known ask-neighbours) nearest to the predicting sample. Each neighbour gets a chance to vote for its class. The predicting sample is assigned to the most voted class [35]. The decision is made based on distance metric. By determining the correct number for k, the optimal classification result can be achieved. The main disadvantage of this method is the need to determine the value of k. Different values of k are usually tested and the k value that gives the least classification error is selected. For the KNN in our application, k values from 3 to 7 were tested and the best performance is obtained for k = 5.

#### 2.5.3. Decision Tree (DT)

DTs provide an effective method of decision making. It is in the form of a tree structure decision support tool that helps to classify the possible outcomes. There are three types of nodes in DT which are root node, leaf node and decision node. Some characteristics of the tree are verified at the root and at each internal node. As a result, the classification of the instances continues down to each internal node based on the value of the characteristic determined in the test. If a leaf, a node with a single incoming edge, is reached then the classification is decided by its class [36]. The main advantage of DT is that it can be visualized easily and is relatively simple to interpret. The DT algorithm can classify both numerical and categorical data while other algorithms can only handle one type of variable. DT has promising results in terms of minimum computation time and thus considered reliable for real-time systems [36]. The C4.5 decision tree was proposed by Ross Quinlan [37] and its rule generation has significantly sped up the training procedure. One of the main drawbacks of DT classification is that over-fitting may occur for a small dataset. For decision trees in our case, default parameters of the Scikit-learn package are adopted based on their good performance. Those parameters are: criterion of quality of split = “gini”; max_depth = None; min_samples_split = 2; min_samples_leaf = 1; min_weight_fraction_leaf = 0; max_leaf_nodes = None; ccp_alpha = 0 (complexity parameter of the pruning); min_impurity_decrease = min_impurity_split = 0.

#### 2.5.4. Support Vector Machine (SVM)

SVM is a very commonly used supervised learning technique that constructs a hyper-plane between the two different classes and tries to maximize the distance of each class from the hyper-plane [38]. It uses various kernel functions (i.e., linear, non-linear, polynomial, radial basis function (RBF) and sigmoid) to maximize the margins between the hyper-planes, which allows solving many complex problems. It has less over fitting problems than other methods. However, the main shortcoming is that SVM needs a large amount of time to train the model if the dataset is large. It is also difficult to choose a proper kernel function for SVM. In our case, the SVC algorithm is used for the SVM classification. SVC stands for C-**s**upport **v**ector **c**lassification and represents the implementation algorithm of SVMs in Scikit-learn and LIBSVM [39]. A poly kernel of a degree = 3 is adopted, and a value of 10 was found to be the best C parameter of the SVC algorithm.

#### 2.5.5. Random Forest (RF)

RF is an ensemble learning method for classification which is based on building multiple DTs to improve the results. Each tree is based on the values of an independently sampled random vector. and all trees in the forest employ a similar distribution. The classification error is a function of the accuracy of distinct trees [38].

An advantage of RF classifier is that it is “over-fitting” resistant. It also has less variance as compared to DT. However, the construction of RF is more time consuming as compared to DT. It also requires more computational resources due to its complexity. Hence, the classification process of RF requires more time to complete [38]. That said, random forest is part of the ensemble methods since it combines the results of several classifiers or estimators. In our implementation, the best number of estimators for the random forest was 100.

#### 2.5.6. Bagging (BG)

Bagging (BG) is a bootstrap aggregation of several classifiers or estimators. It is considered as an ensemble meta-estimator. Bagging grows many estimators, and each individual estimator is considered as a weak learner. However, when multiple estimators are combined, they are considered as strong learners. Thus, multiple classifiers are allowed to fit the training data so that any bias, such as over-fitting, and can be dealt with by using the ensemble of the classifiers resulting in good classification results. This method works well with imbalanced data [38], by reducing the variance and hence overfitting. In our case, 10 SVC estimators were chosen for the bagging method. The SVM/SVC estimator is adopted since the high observed performance of that algorithm as further described in the results section.

### 2.6. Performance Evaluation Metrics

#### 2.6.1. Compression Ratio

It estimates the performance of the proposed system in terms of the reduction in quantity of information which is required to be transmitted and classified. The comparison is achieved with the classical method of transmitting acquired ECG signals to the cloud without making any dimension reduction and features extraction. In the classical situation, every element of the dataset $P_{r}$ is encoded using 11-Bit resolution [28]. Here, $P_{r}$ is the dataset, which has to be transferred and categorized for the classical case. In the proposed solution each element in the selected features dataset P2 is encoded using 11-Bit resolution as well. Thus, the compression ratio in Bits, $R_{COMP}$, may be computed using Equation (4):

(4) $$R_{COMP}=\frac{P_{r}}{P2}$$

#### 2.6.2. Computational Complexity

It allows comparing the developed system performances with the fix-rate counter equals in terms of the count of necessary basic operations like multiplications and additions [40]. The complexity of the processing chain at the front end is analysed in depth. In order to accomplish the classification task, the difficulty of cloud-based classification is evaluated by examining the time delay.

**Figure 4:**
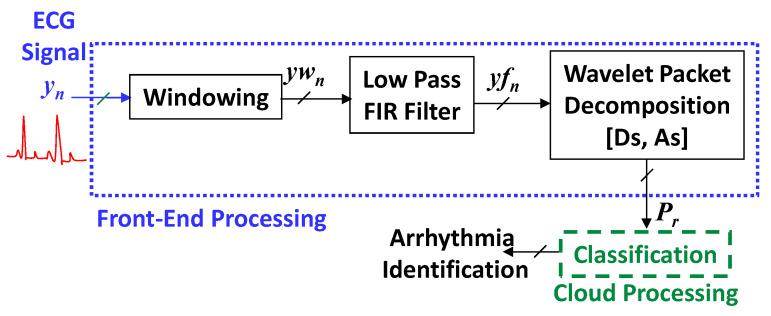
The classical fixed-rate approach block diagram.

For the case of fix-rate counterpart the processing principle is shown in Figure 4. In This case, $y_{n}$ is also windowed in to cardiac pulses of 0.9 s in length, each consist of $N_{r}\;$ = 324 samples. It is attained by using a rectangular window function [30]. Compared with operations such as additions and multiplications, the processing cost of this procedure is negligible [40]. Onward, each window is denoised by employing an FIR filter, operates at $F_{S}=360$ Hz. The computational complexity of a $K_{r}$ order FIR filter, while computing an output sample, is $K_{r}-1$ addition operations and $K_{r}$ multiplication operations [30]. Then the denoising task complexity for $N_{r}$ samples could be given by Equation (5):

(5) $$C_{FR-FIR}=\underset{Additions}{\underbrace{(K_{r}-1)\times N_{r}}}+\underset{Multiplications}{\underbrace{K_{r}\times N_{r}}}$$

**Figure 5:**
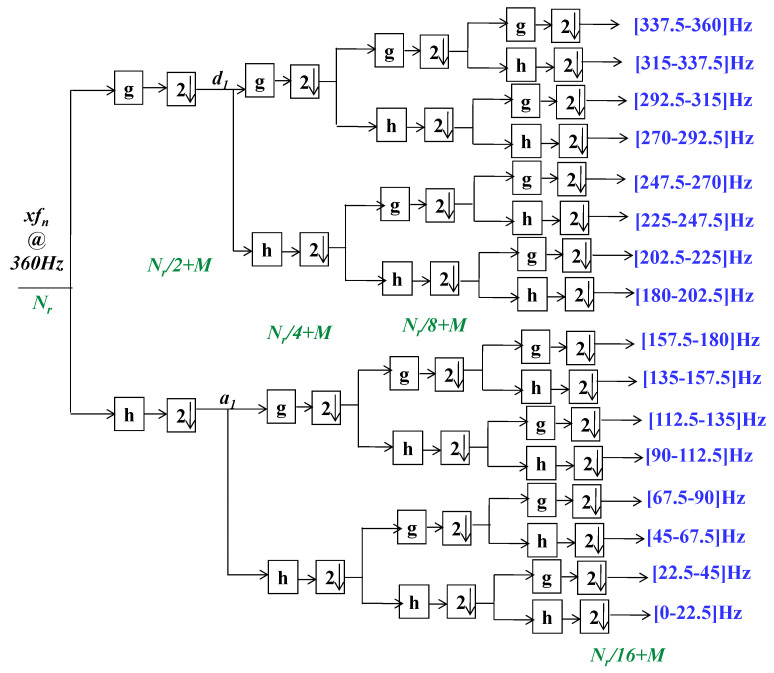
The classical wavelet packet decomposition scheme.

Each denoised segment could be further divided into subbands by means of the 4th level Daubechies wavelet packet decomposition. (cf. Figure 5). Let $K_{wd}=M+1$ be the order of FIR half-band filters, adopted during the subband decomposition task. Consequently, the derivation of computational complexity, $C_{FR-WD}$, of this process could be illustrated as indicated by Figure 5. It demonstrates that, at the first step, two $K_{wd}$ order half-band filters are denoising 2. $N_{r}$ samples. The filtered signals are then decimated by a factor of two. By collecting the first sample, zero index, and then every indexed sample, it is realized. It decreases by a factor of two the decimated signal sampling rate. The complexity of this decimation task is insignificant because no multiplication, addition, or split operations are required [30,40]. In continuity of Equation (5), it is possible to define the complexity of the first stage of decomposition as: $\underset{Additions}{\underbrace{(K_{wd}-1)\times 2\times N_{r}}}+\underset{Multiplications}{\underbrace{K_{wd}\times 2\times N_{r}}}$. Similarly, by counting the number of multiplications and additions at 2nd, 3rd and 4th stages of decomposition, the total computational complexity of the adopted wavelet decomposition task could be described by Equation (6):

(6) $$C_{FR-WD}=\underset{Additions}{\underbrace{\left(K_{wd}-1\right)\times \left(8\times N_{r}+28M\right)}}+\underset{Multiplications}{\underbrace{K_{wd}\times \left(8\times N_{r}+28M\right)}}$$

For the implemented system, $y_{n}$ is initially subsampled and then $yd_{n}$ is segmented into QRS complexes. This segmentation operation on average performs 80 magnitude comparisons per segment. Onward this segment is denoised by means of a FIR filter, operates at $Fs1=\frac{Fs}{2}=180\;H$z. $N_{d}\;$ *=* 60 is the count of samples per segment, $ys_{n}$. In this case, the filter is designed for similar design parameters, used in the conventional case, such as cut-off frequency and transition band. Therefore, its count of coefficients is half as compared to the one designed to operate at Fs = 360 Hz. Let the order of denoising filter is K  in this case. Then it is related to $K_{r}$ as: $K=0.5\times K_{r}$. Following Equation (5), the complexity of denoising $N_{d}$ samples by the K order FIR filter can be expressed by Equation (7). In order to keep the complexity of the proposed system coherent with the traditional one, it is assumed that the arithmetic cost of a comparison is equal to that of an addition. Therefore this count of comparisons is added in the count of additions in Equation (7):

(7) $$C_{P-FIR}=\underset{Additions}{\underbrace{\left(K-1\right)\times N_{d}+80}}+\underset{Multiplications}{\underbrace{K\times N_{d}}}$$

For the devised solution, the denoised signal, $x_{n}$ is further subsampled. After this 2nd step of subsampling, every denoised segment is made of N=30 samples. If $K_{wd}$ is the order of half-band FIR filters, used during decomposition process of the subbands, then according to Equation (7) the computational complexity of this task, $C_{P-WD}$, can be formulated mathematically by Equation (8):

(8) $$C_{P-WD}=\underset{Additions}{\underbrace{\left(K_{wd}-1\right)\times \left(3.75\times N+8M\right)}}+\underset{Multiplications}{\underbrace{K_{wd}\times \left(3.75\times N+8M\right)}}$$

The total computational complexity, $C_{FR-Total}$, for the fix-rate front-end processing chain, per window $yw_{n}$, is derived by combining the operations count which are presented in Equations (5) and (6). $C_{FR-Total}$ is given by Equation (9):

(9) $$C_{FR-Total}=\underset{Additions}{\underbrace{(K_{r}-1)\times N_{r}+\left(K_{wd}-1\right)\times \left(8\times N_{r}+28M\right)}}+\underset{Multiplications}{\underbrace{K_{r}\times N_{r}+K_{wd}\times \left(8\times N_{r}+28M\right)}}.$$

The total computational complexity, $C_{P-Total}$, for the proposed front-end ECG processing chain, per segment $xs_{n}$, is derived by combining the operations count which are presented in Equations (7) and (8). $C_{P-Total}$ is given by Equation (10):

(10) $$C_{P-Total}=\underset{Additions}{\underbrace{\left(K-1\right)\times Nd+80+\left(K_{wd}-1\right)\times \left(3.75\times N+8M\right)}}+\underset{Multiplications}{\underbrace{K\times Nd+K_{wd}\times \left(3.75\times N+8M\right)}}$$

#### 2.6.3. Reconstruction Error

It allows quantifying the effectiveness of the adopted subsampling-based compression methodology. In this context, recalculated versions of both compressed signals, $yw_{n}$ and $x_{n}$ are produced using the 4th order cubic spline interpolation (CSI) [30]. Let $yw_{n}^{\sim }=yd_{\frac{n}{2}}$ and $x_{n}^{\sim }=xd_{\frac{n}{2}}$ be, respectively, the recalculated versions of $yw_{n}$ and $x_{n}$, then the reconstruction error is calculated using MSE (mean square error). The MSE for each considered instance is computed using Equations (11) and (12). In this context, *MSE*1 and *MSE*2 represent the Mean-Square-Errors for the 1st and 2nd levels of the decimations, respectively.

(11) $$MSE1=\frac{\sum_{n=1}^{N_{r}}{\left(yw_{n}-yw_{n}^{\sim }\right)}^{2}}{N_{r}}$$

(12) $$MSE2=\frac{\sum_{n=1}^{M=N_{d}}{\left(x_{n}-x_{n}^{\sim }\right)}^{2}}{N_{d}}.\;$$

#### 2.6.4. Classification Evaluation

**Figure 6:**
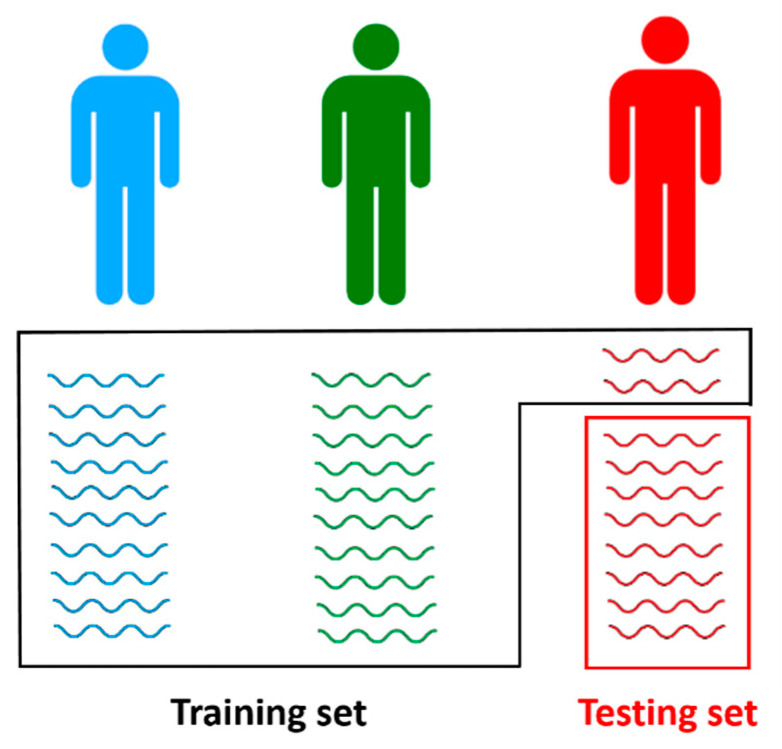
Example of one-fold (iteration) of the partial blind testing protocol. In this fold, the training set is composed of the first two subjects’ samples (blue and green subjects) plus a limited number of samples from the testing subject (red subject). The latter small part extracted from the testing subject is important for calibration purpose.

In terms of hardware simplicity, compression and transmission efficiency improvements, the suggested solution appears promising. In terms of accuracy, however, it can lag. Thus, in terms of its classification precision, the effectiveness of the entire system was investigated. The cross-validation model has been widely used in the literature [37,38] to prevent any bias in evaluating the classification output due to the small dataset. In this study, two approaches of cross-validation are implemented. The first approach represents a classic 5CV. This type of approach is largely applied in previous works since its advantages in treating over fitting issues [6,7,22,24,41]. The second approach relies on what we call “partial blind testing” as shown in Figure 6. In this approach, the dataset samples are split respecting different subjects between training and testing sets. Hence, samples used in testing are mostly extracted from subjects not included in the training set. However, some samples from testing subjects were intentionally injected into the training set. These samples do not exceed 29% of the available testing samples and of course were discarded from the testing set. This operation is mandatory to homogenize signals and plays a calibration role in helping the system to adapt to new subject’s signals. Note that cross-validation is also applied for the “partial blind testing” protocol. The cross-validation folds are thus equal to the number of subjects used in each dataset.

For classification evaluation, the following assessment criteria were used to prevent any prejudice in the results.

*Accuracy (Acc)*

Accuracy is the number of marks that have been classified correctly. Let *TP*, *TN*, *FP* and *FN*, respectively, in the expected labels, denote true positives, true negatives, false positives and false negatives. The mathematical model for Acc is calculated using Equation (13). The measurement of Acc results in a range between zero and one, with higher values indicating improved efficiency.

(13) $$Accuracy=\frac{TP\;+\;TN}{TP\;+\;TN\;+\;FP\;+\;FN}$$

*F-Measure (F1)*

The F-measure (F1) tries to balance the recall and precision values. We typically talk about an F-measure micro (taking into account class sizes) or macro (without taking class size into consideration). However, because all groups in the considered case had the same data size, we simply employ the *F*-measure. Formally, the *F*-measure is given by Equation (14), where *precision* = $\frac{TP}{\left(TP+FP\right)}$ and *recall* = $\frac{TP}{\left(TP+FN\right)}$:

(14) $$F=\frac{2*precision*recall}{precision+recall}$$

When computing the F1-measure in Scikit-learn, we adopted the micro-F1 variant. The “micro” variant globally computes rates by counting the total true positives, total false positives and total false negatives. In the multilabel case, when calculating the micro-precision (TP/TP + FP), the total number of false positives corresponds actually to the total number of classification errors. Similarly, when calculating the micro-recall (TP/TP + FN), the total number of false negatives also correspond to the total number of classification errors. As a result, the micro-precision is found to be equal to micro-recall, equal to micro-F1 and equal to accuracy. On that basis, we chose to omit F1-score results and present only accuracy rates.

*Kappa Index (Kappa)*

The kappa is a commonly used statistical method for determining the agreement between the two effects of clustering. Typically, it is considered more rigorous than simple precision since it takes into consideration the probability of agreement by chance. The most common version is the *kappa* measure of Cohen, which is expressed mathematically by Equation (15):

(15) $$kappa=1-\frac{1-p_{0}}{1-p_{e}}.\;$$

where *p*_0_ is the percentage of agreement, close to precision, between the expected and real labels, and *p_e_* is the theoretical probability of a random occurrence of such a contract as expressed by Equation (16):

(16) $$p_{e}=\frac{\left(TP+TN\right)\left(TP+FN\right)\;+\;\left(FP+TN\right)\left(FP+FN\right)}{{\left(TP+TN+FP+FN\right)}^{2}}$$

An ideal classification corresponds to *kappa* = 1, and in the case the classification is simply induced by chance then we have *kappa* = 0.

*Receiver Operating Characteristics (ROC) curves*

The receiver operating characteristics (ROC) curves reflect the efficiency of a classifier. It’s a two-dimensional graph in which the false positive rate is plotted on the *x* axis and the true positive rate is plotted on the *y* axis. A high area under the ROC curve, referred as AUC, better is the ability of the model for correct classification. Hence, the AUC ranges in value from 0 to 1. A model whose predictions are all wrong has an AUC of 0, whereas a model whose all predictions are correct has an AUC of 1.0. The ROC line depends on the specifications of the test dataset under investigation. By using the cross-validation methodology, this sample dependence can be minimized [42].

## 3. Results

**Figure 7:**
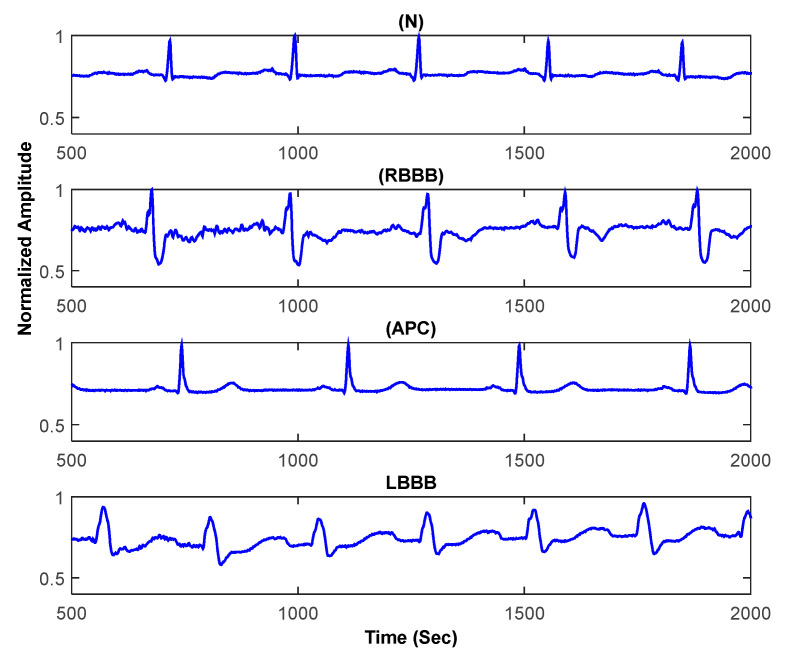
Examples of pre-windowed ECG signals.

**Figure 8:**
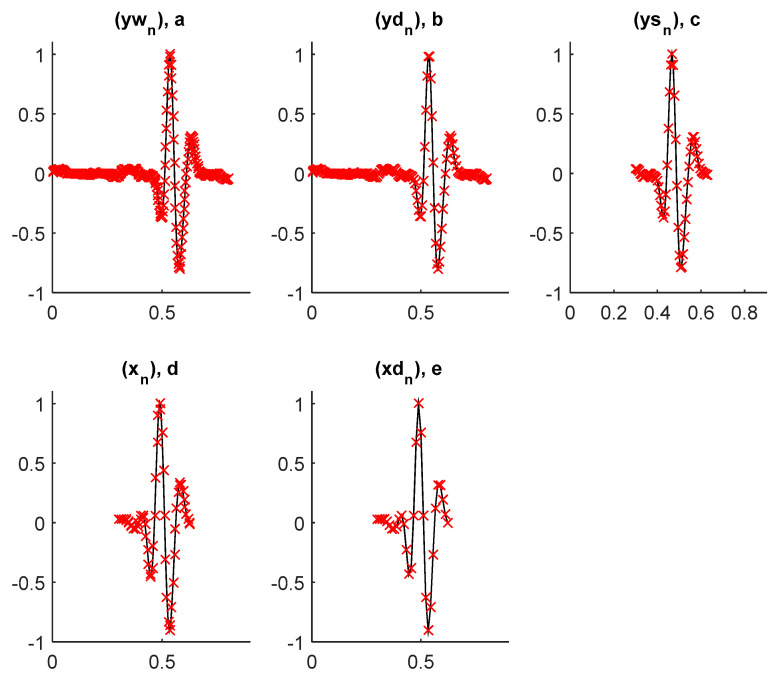
Different front-end processing stages on a RBBB heartbeat, where the *y*-axis is the normalized amplitude and *x*-axis is the time in seconds (**a**) an example of the windowed signal $yw_{n}$; (**b**) the subsampled signal $yd_{n}$; (**c**) $ys_{n}$ obtained after the segmentation of QRS complex from $yd_{n}$; (**d**) denoised signal $x_{n}$; (**e**) $xd_{n}$ obtain after $x_{n}$ is subsampled with a factor of 2.

The performance of suggested method is studied for the MIT-BIH arrhythmias Database [28]. Figure 7 displays examples of pre-windowed signals of the considered arrhythmias. The incoming signal, $y_{n}$ is divided in 0.9-s fix-length windows. An example of the windowed signal $yw_{n}$ is shown in Figure 8a. It is a portion of record number 124 and is a cardiac impulse from the class RBBB [28]. $yw_{n}$ is subsampled with a factor of 2. The subsampled signal $yd_{n}$ is shown in Figure 8b. By comparing Figure 8a,b it is observable that the first stage of decimation only removes the redundant information from $yw_{n}$ without introducing evident modifications in it. Onward, QRS complex is segmented from $yd_{n}$ to obtain $ys_{n}$. $ys_{n}$ is shown in Figure 8c. Furthermore, $ys_{n}$ is denoised by using a prior-designed 45th order FIR low-pass filter with the cut-off frequency of $F_{C}\;$ = 40 Hz. The denoising enhances the signal-to-noise ratio (SNR) of the intended signal which results in a better features extraction and classification [6]. The denoised signal $x_{n}$ is shown in Figure 8d. Before wavelet decomposition, $x_{n}$ is further subsampled with a factor of 2 to obtain $xd_{n}$, which is shown in Figure 8e. This second stage of decimation further removes redundancy from $x_{n}$ and allows achieving the wavelet decomposition operation with reduced computational complexity [7].

The reconstruction errors for both subsampling based compression stages are computed by using Equations (11) and (12). In this study four ECG classes are considered, each with 510 segments or instances. Mean reconstruction errors obtained for 510 instances of each class are summarized in Table 1. It shows that the average *MSE*1 for all classes is 32.514 × 10^−6^ V^2^ and the average *MSE2* for all classes is 1.256 × 10^−6^ V^2^.

**Figure 9:**
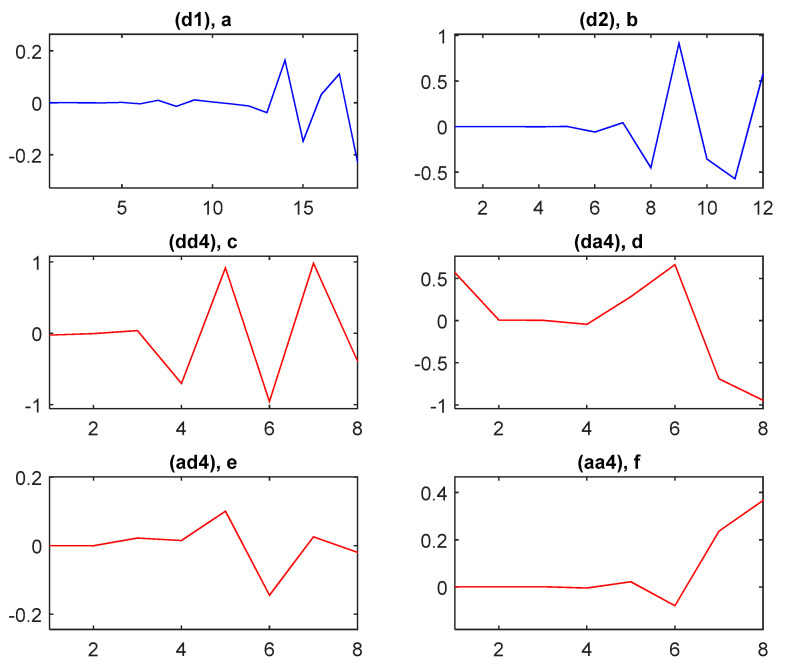
Example of subband coefficients of a RBBB QRS complex segment, where the *y*-axis is the normalized amplitude and *x*-axis is the number of coefficients. (**a**) shows coefficients of the subband d1, (**b**) shows coefficients of the subband d2, (**c**) shows coefficients of the subband dd4, (**d**) shows coefficients of the subband da4, (**e**) shows coefficients of the subband ad4, and (**f**) shows coefficients of the subband aa4.

After the second level of subsampling, each segment, $\;xd_{n}$, is decomposed into subbands by using a 4th level proposed wavelet decomposition scheme. Fourth-order half-band FIR analysis filters are employed in this framework. This allows extracting the pertinent features from $xd_{n}$. Examples of subband coefficients, obtained by applying the proposed wavelet decomposition scheme for the aforementioned cardiac impulse from the class RBBB, are shown in Figure 9.

The online subsampling results in signal denoising, decomposition and dimension reduction with a reduced computational cost when compared with the fix-rate counterparts [12,14,15,16,17,22,23]. In fix-rate counterparts the x(t) is acquired and processed at $F_{S}=360\;\mathrm{Hz}$. In this case, $yw_{n}$ is denoised by using a prior-designed 91st-order FIR low-pass filter with cut-off frequencies of $F_{C}\;$ *=* 40 Hz. For each window, it renders $N_{r}\;$ = 324 samples for a given time-length of 0.9-s. Each denoised segment is decomposed into subbands by using a 4th-order half-band FIR analysis filters based4th level wavelet packet decomposition.

Computational effectiveness of the suggested processing chain over the fix-rate counterpart is computed by using Equations (9) and (10). Compared to the fix-rate counterparts, it brings the 12.2-fold 12.4-fold reduction, respectively, in terms of count of additions and multiplications of the suggested solution.

From Figure 1, it is evident that the proposed system operates at multirates. It is attained by effectively incorporating two cascaded stages of decimation in the system. Each stage introduces a subsampling with a factor of 2. Additionally, QRS complex segmentation allows focusing on the concerned cardiac pulse portion. After suggested wavelet decomposition scheme we obtained 62 features per instance and results in a feature set *P*1 = 2040 × 62 for all considered four-class arrhythmia instances. After subband features selection module, the selected features set *P*2 = 2040 × 32 is obtained. In the traditional fix-rate counterpart without subbands selection, the 4th level wavelet packet decomposition results 416 subband coefficients per instance. Therefore, it results in a feature set of *P_r_* = 2040 × 416 dimension. By using Equation (4), the compression gain of the suggested method is computed. It brings the 13-fold diminishing in the volume of information to be transmitted and processed by the post cloud server-based application compared to the fix-rate classical counterparts.

To demonstrate the benefit of proposed dimension reduction approach, the classification results obtained for the case of features set P2 are compared with ones obtained for the features set P1. Six classifiers are applied to our datasets settings using the 5CV and the partial blind testing protocol (partial blind). These classifiers are based on ANN, KNN, DT, SVM, RF and BAG. All results are detailed in the following tables.

Table 2, Table 3 and Table 4 present all evaluation metrics showing high-performance rates for all proposed classifiers. For the 5-fold cross validation protocol, the best accuracy rate was **97.35%** obtained by the RF classifier for P1. The confusion matrix of that result is presented in Table 5. Similarly, the RF classifier obtained the best Kappa rate (**96.47%**) and the best AUC rate (**99.86%**).

Comparing the two datasets results (P1 and P2), the full setting (P1) shows to give slightly higher performances almost for all classifiers and metrics. For instance, for P2, the best accuracy rate was **97.06%** obtained by the SVM classifier. The corresponding confusion matrix is shown in Table 6. The best kappa rate was **96.07%** obtained by SVM, and the best AUC rate was **99.77%** obtained by RF. These observed slight differences between the two settings results consolidate the robustness of the proposed dimension reduction approach.

Moreover, for the 5CV protocol, besides RF and SVM which seem to be respectively the best classifiers, the BAG method has led also to quite interesting rates. For instance, for the full setting, the BAG classifier results were **96.96%**, **95.94%** and **99.79%**, respectively for accuracy, kappa and AUC rates.

For the partial blind, the best accuracy rate was **92.99%** obtained by the RF classifier for P1. The confusion matrix of that result is presented in Table 7. Furthermore, the RF classifier obtained the best Kappa rate (**90.65%**) and the best AUC rate (**99.51%**).

Similarly to the 5CV protocol, when comparing the two datasets results (P1 and P2) within the partial blind, the full setting (P1) presents slightly better performances and demonstrates the efficiency of the feature reduction approach. For instance, for P2, the best accuracy rate was **92.08%** obtained by the ANN classifier. The corresponding confusion matrix is shown in Table 8. The best Kappa rate was **89.44%** obtained by ANN and the best AUC rate was **98.84%** obtained by RF. Overall, besides RF and ANN classifiers, the SVM and the BAG classifiers have also shown high performances for all evaluation metrics in the Partial Blind.

Considering the two different protocols, although the 5CV protocol shows to secure higher rates (**92.08%** vs. **97.06%** for accuracy, **89.44%** vs. **96.07%** for kappa and **98.84%** vs. **99.77%** for AUC), the partial blind still gives high-performance results and presents a solid approach for implementing efficient arrhythmia classification systems.

Moreover, confusion matrices shown in Table 5, Table 6, Table 7 and Table 8 further emphasize the effectiveness of our models for all classes and both protocols. However, among all classes, the APC class in particular seems to have higher false negative predictions. For P1-5CV and P2-5CV, among the 510 APC instances 24 and 22 were respectively predicted as normal (N). However, no particular issue is observed when predicting the N class for the P1-5CV case. For the P2-5CV case, among the 510 Normal (N) instances10 were respectively predicted as APC. For P1-partial blind and P2-partial blind, among the 360 APC instances 26 and 28 were respectively predicted as normal (N), and reciprocally 21 and 40 from the 360 normal samples, were respectively predicted as APC. This confusion between these two particular classes suggests investigating further feature extraction methods in future work in order to get new features having more discriminated representation especially for these two classes.

To summarize, considering the two different protocols, although the 5CV protocol shows to secure higher rates (97.35% (±0.72) vs. 92.99% (±1.39) for P1 and 97.06% (±0.89) vs. 92.08% (±2.30) for P2), the partial blind still gives high-performance results and present a solid approach for implementing efficient arrhythmia classification systems. In addition, as expected, higher standard deviations were observed for the Partial Blind testing protocol, especially for the accuracy metric. This is explained by the intrinsic nature of the used protocol in preparing training and testing data. In fact, in the 5CV protocol, data were randomly divided resulting in very close and homogenous splits. In the partial blind, splits were structured based on different subjects resulting in sparser and more heterogeneous splits, which affected the variance of the classification results.

More generally, the decrease of performance observed for the proposed, partial blind, protocol can be explained by two facts. On the first hand, for the classic cross validation protocol, patients’ samples were pseudo-randomly distributed between training and testing sets, which makes the system less sensitive to underfitting issues. On the other hand, for the partial blind, considering a testing patient, just a few samples of its instants were included in the training process, which makes the system more sensitive to overfitting issues.

Therefore, between classic cross-validation and a total blind testing protocol, the “partial blind” represents a good compromise in terms of efficiency and generalization. Concretely, the “Partial Blind” can be implemented in a real-world application with a minor intervention of medical experts. Actually, when the system is configured for a new patient, the cardiologist should manually label few samples in order to calibrate the machine for that new patient. This operation is required only once especially during the first examination of the patient and onward system can operate autonomously.

From the above discussion and for all studied cases, we can conclude that RF, SVM, BAG, and ANN classifiers show to be the best arrhythmia classification methods. Overall, the presented evaluation metrics demonstrate the effectiveness of multirate processing and feature extraction processes as well as the relevance of our classification protocols and approaches.

## 4. Discussion

The developed solution’s attractive features are evident from the results described in Section 3. It is realized by intelligently achieving the multirate processing of ECG signals, effective decomposition of subbands and the selection of attributes based on frequency content. Online decimation prevents the processing of unwanted data points and enables denoising of the signal with a lower order filter compared to the one adopted for fix-rate counterparts. According to Zhang et al. [43], the sub-bands derived by any level of wavelet decomposition could be considered functions of the incoming signal sampling frequency. $Fs2=\frac{FS1}{2}\;$ = 90 Hz presents the denoised and decimated segment, $xd_{n}$, frequency contents in a better fashion than $F_{S}$. Therefore, compared to the fix-rate counterparts [12,14,15,16,17,22,23], it offers better performance in terms of subband focus and computational complexity while performing the subbands decomposition. All in all, the planned front-end processing chain achieves more than 12-fold benefit in additions and multiplications compared to the fix-rate counterparts in the studied case.

The error caused by the adopted subsampling processes is measured in terms of RMSD. It is obvious from Table 1 that the compression based on subsampling causes only slight alterations in the original signal and does not create any major loss of information.

In the traditional case, the standard 4th level decomposition based on the wavelet packet reproduces 416 subband coefficients. In our case, its dimensionality is decreased to 32 coefficients after selected subband coefficients are extracted. Indeed the selection of features offers many benefits. Firstly, it enhances the bandwidth usage efficiency, compression ratio and transmission activity. In the considered case it resulted in $R_{COMP}\;$ = 13-fold for each instance. Compared to traditional fix-rate equivalents, this aptitudes the same decreasing factor in data transmission operation, power consumption and bandwidth utilization. In addition, the processing of 13-fold lower amount of data on the cloud side promises a comparable advantage in terms of resource utilization during the classification task. In addition, minor variations were found as compared to the different settings (i.e., complete features set  P1 andreduced features set P2), consolidating the robustness of the proposed feature selection method. For most of the intended classifiers, such as SVM, RF, ANN and BAG, the attained classification accuracy is more than or at least equal to 95.83% for the 5CV case and 90.35% for the partial blind. The highest attained accuracies for the case of P2 dataset are, respectively, 0.29% and 0.91% less than the highest attained accuracy for the case of P1 dataset for 5CV and Partial Blind cases. It confirms the effectiveness of proposed frequency content based subbands coefficients selection. It not only permits the post classifiers to attain a high accuracy but also promises a significant reduction in the latency and computational load of classification process because it has to deal with a lower dimension features set P2 in place of P1.

The suggested methodology is novel, and it is not straightforward to compare it with current state-of-the-art methods since most of the experiments are focused on a fix-rate concept of processing. In addition, diverse classification and ECG signal processing techniques are used. However, a comparison between key previous studies using the same ECG dataset is presented. The highest accuracies of classification for all considered studies are summarized and compared in Table 9. It assures that, compared to previous equals, the suggested solution secures an equivalent or better classification performance [12,14,15,16,17,22,23].

In carrying out the signal denoising and subband decomposition, the main benefit of the proposed method over the previous fix-rate ones is to eliminate the unnecessary samples and operations [12,14,15,16,17,22,23]. Compared to previous research, it secures real-time compression and computational performance. In addition, the selection of content dependent subbands promises an efficient data transfer with decreased post classification complexity and latency. In addition, relative to the previous state of the art solutions, the suggested method secures comparable or greater classification accuracy. Due to the computing efficiency and lower size of transmitted data while achieving high arrhythmia classification accuracy, it is particularly beneficial for the realization of low-power ECG wearable gadgets with cloud-assisted diagnosis.

## 5. Conclusions

In this paper a novel multirate processing chain is proposed for computationally efficient arrhythmia classification. The proposed method avoids the processing of redundant information while allowing the reconstruction of the signal with adequate precision. Hence the computational complexity of the proposed solution is less than the fix-rate methods and achieves more than 12-fold overall gain in additions and multiplications for the proposed front-end processing chain. The proposed framework also benefits from the content-based selection of subbands and has achieved a 13-fold compression gain over the traditional methods. Results have shown that the dimension reduction method is also beneficial for the post cloud-based classification module. It promises to augment its computational effectiveness without significantly compromising the accuracy. For the studied cases, the highest classification accuracy of more than 97% is achieved, which is equivalent, and in some cases better, than the existing contemporary solutions. The major advantage of this approach is its compression and computational benefits. Hence, the proposed multirate processing chain can be incorporated in cloud-based mobile healthcare applications. A future task is to enlarge the range of each tested hyperparameter while searching for the best configuration of considered classifiers. Hopefully, it will improve the performance of classification. Another axis to explore is the possibility of incorporating the deep learning algorithms in the suggested approach.

## Figures and Tables

**Table 1:** Mean reconstruction errors.

Class	MSE1 ×(10^−6^) V^2^	MSE2 ×(10^−6^) V^2^	For All Classes MSE1 ×(10^−6^)V^2^	For All Classes MSE2 ×(10^−6^)V^2^
N	6.837	2.173	32.514	1.256
RBBB	82.765	1.832		
APC	25.691	0.202		
LBBB	14.762	0.816		

**Table 2:** Accuracy of different classifiers for arrhythmia classification in the two proposed protocols.

Accuracy				
Protocol	5CV		Partial Blind	
Classifier/Dataset	P1	P2	P1	P2
ANN	96.62 (±0.81)	95.83 (±1.18)	91.60 (±3.14)	**92.08** (±2.30)
KNN	96.37 (±0.57)	96.27 (±0.42)	91.32 (±2.35)	90.07 (±2.15)
DT	95.34 (±0.79)	93.92 (±0.95)	87.08 (±4.34)	85.00 (±4.09)
SVM	96.76 (±0.84)	**97.06** (±0.89)	91.67 (±1.48)	90.62 (±2.67)
RF	**97.35** (±0.72)	96.91 (±0.50)	**92.99** (±1.39)	91.60 (±0.20)
BAG	96.96 (±0.92)	96.52 (±1.32)	90.00 (±3.58)	90.35 (±1.75)

**Table 3:** Kappa results of different classifiers for arrhythmia classification in the two proposed protocols.

Kappa				
Protocol	5CV		Partial Blind	
Classifier/Dataset	P1	P2	P1	P2
ANN	95.48 (±1.08)	94.43 (±1.58)	88.80 (±4.18)	**89.44** (±3.07)
KNN	95.16 (±0.76)	95.03 (±0.56)	88.43 (±3.13)	86.76 (±2.87)
DT	93.78 (±1.05)	91.89 (±1.27)	82.78 (±5.79)	80.00 (±5.46)
SVM	95.68 (±1.12)	**96.07** (±1.19)	88.89 (±1.98)	87.50 (±3.56)
RF	**96.47** (±0.96)	95.88 (±0.68)	**90.65** (±1.85)	88.80 (±0.26)
BAG	95.94 (±1.24)	95.35 (±1.76)	86.67 (±4.78)	87.13 (±2.33)

**Table 4:** AUC results of different classifiers for arrhythmia classification in the two proposed protocols.

AUC				
Protocol	5CV		Partial Blind	
Classifier/Dataset	P1	P2	P1	P2
ANN	99.67 (±0.15)	99.50 (±0.19)	98.83 (±0.66)	97.90 (±0.47)
KNN	99.37 (±0.22)	99.21 (±0.37)	98.30 (±0.47)	97.78 (±0.66)
DT	96.90 (±0.55)	95.99 (±0.60)	91.39 (±2.90)	90.00 (±2.73)
SVM	99.55 (±0.23)	99.49 (±0.26)	98.88 (±0.35)	97.62 (±0.58)
RF	**99.86** (±0.08)	**99.77** (±0.10)	**99.51** (±0.24)	**98.84** (±0.17)
BAG	99.79 (±0.09)	99.69 (±0.16)	99.15 (±0.55)	98.33 (±0.29)

**Table 5:** Confusion matrix for the RF classifier applied to the P1 dataset within the 5CV protocol (Accuracy = 97.35%).

RF (P1−5CV)		Predicted			
		N	RBBB	APC	LBBB
Actual	N	498	1	4	7
	RBBB	3	501	0	6
	APC	24	1	481	4
	LBBB	3	0	1	506

**Table 6:** Confusion matrix for the SVM classifier applied to the P2 dataset withinthe 5CV protocol (Accuracy = 97.06%).

SVM (P2−5CV)		Predicted			
		N	RBBB	APC	LBBB
Actual	N	494	1	10	5
	RBBB	2	504	0	4
	APC	22	3	482	3
	LBBB	5	2	3	500

**Table 7:** Confusion matrix for the RF classifier applied to the P1 dataset within the partial blind testing protocol (accuracy = 92.99%).

RF (P1−Partial Blind)		Predicted			
		N	RBBB	APC	LBBB
Actual	N	333	3	21	3
	RBBB	9	332	14	5
	APC	26	1	327	6
	LBBB	1	0	12	347

**Table 8:** Confusion matrix for the ANN classifier applied to the P2 dataset within the partial blind testing protocol (accuracy = 92.08%).

ANN (P2−Partial Blind)		Predicted			
		N	RBBB	APC	LBBB
Actual	N	315	4	40	1
	RBBB	7	338	10	5
	APC	28	0	328	4
	LBBB	1	9	5	345

**Table 9:** Comparison with state-of-the-art methods.

Study	Features Extraction	Classification Method	No. of Classes	Accuracy (%)
[12]	Tunable Q-wavelet Transform (TQWT)	SVM (36% training-64% testing split, without blind testing)	8	99.27
[14]	DWT + Fuzzy and Renyi Entropy + Fractal Dimension	KNN (CV)	5	98.1
[15]	Wavelet Packet Entropy (WPE)	RF (Inter-Patient Scheme with CV)	5	94.61
[16]	Discrete Wavelet Transform (DWT)	Probabilistic Neural Network (PNN) (50% training-50% testing split, without blind testing)	8	92.75
[17]	Short Time Fourier Transform (STFT)	Convolutional Neural Network (CNN) (80%training-20%testing split, without blind testing)	5	99.0
[22]	DWT + Temporal + Morphological	SVM (CV)	4	98.4
[23]	DWT	Long Short Term Memory (LSTM) (60% training-20% validation-20% testing split, without blind testing)	5	99.4
[24]	LSTM-based auto-encoder (AE) network	SVM (CV)	5	99.45
[25]	DWT + RR Interval + Teager Energy Operator	ANN (CV)	5	99.75
This Study	Specific Wavelet Decomposition Scheme + Content based subbands selection, [P2]	ANN (Partial Blind)	4	92.09
	Specific Wavelet Decomposition Scheme + Content based subbands selection, [P2]	SVM (5CV)	4	97.06

## Data Availability

The data, studied in this research, is publically available under the link, mentioned in [29].
